# Human heart disease: lessons from human pluripotent stem cell-derived cardiomyocytes

**DOI:** 10.1007/s00018-017-2546-5

**Published:** 2017-06-01

**Authors:** E. Giacomelli, C. L. Mummery, M. Bellin

**Affiliations:** 1 Department of Anatomy and Embryology, Leiden University Medical Center, Einthovenweg 20, 2333 ZC Leiden, The Netherlands; 2 Department of Applied Stem Cell Technologies, University of Twente, Building Zuidhorst, 7500 AE Enschede, The Netherlands

**Keywords:** Human pluripotent stem cell-derived cardiomyocytes, Disease modeling, Cardiac disease, Cardiovascular disease, Safety pharmacology, Drug screening, Cardiac arrhythmia, Cardiomyopathy

## Abstract

Technical advances in generating and phenotyping cardiomyocytes from human pluripotent stem cells (hPSC-CMs) are now driving their wider acceptance as in vitro models to understand human heart disease and discover therapeutic targets that may lead to new compounds for clinical use. Current literature clearly shows that hPSC-CMs recapitulate many molecular, cellular, and functional aspects of human heart pathophysiology and their responses to cardioactive drugs. Here, we provide a comprehensive overview of hPSC-CMs models that have been described to date and highlight their most recent and remarkable contributions to research on cardiovascular diseases and disorders with cardiac traits. We conclude discussing immediate challenges, limitations, and emerging solutions.

## Introduction

Human embryonic stem cells, derived from the early human embryos, and human-induced pluripotent stem cells, derived by reprogramming somatic cells (hESCs and hiPSCs, respectively, and collectively called hPSCs) can self-renew and differentiate into all cell types of the human body, including cardiomyocytes [1–3]. They have potential applications in regenerative medicine but are also becoming a useful tool in cardiovascular research. Most particularly, they offer new opportunities to develop in vitro models of human cardiac development and cardiovascular diseases, as they are able to capture much of the normal and pathological physiology of the human heart, including aspects of congenital defects. In addition, hPSC-derived cardiomyocytes (hPSC-CMs) may be used in cardiac safety pharmacology, drug screening, and drug discovery, to predict the effects of candidate drugs and new compounds and to identify key target pathways in disease. Whilst hESCs can now readily be engineered to carry specific disease mutations, the derivation of hiPSCs from virtually any patient of interest offers some advantages over hESCs for disease modeling, since hiPSCs incorporate individual complex genetic backgrounds of the patients from which they were originated. For this reason, expectations are high on their contribution to precision medicine where the goal is to prevent disease development and find personalized treatments that take genetic variability of patients into account [4].

In this review, we provide comprehensive coverage of hPSC models of human heart disease.

## Generation of hiPSCs and hESCs for cardiac disease modeling

The need for more robust cell models for human disease, including cardiovascular disorders, has led to increasing interest in hPSCs.

hESCs were the first human pluripotent stem cells described. They were derived from the inner cell mass of blastocyst-stage embryos in 1998 by Thomson [1]. These cells could differentiate toward cell lineages of all three germ layers yet to be maintained in a state of self-renewal indefinitely in their undifferentiated state. Multiple hESC lines have been used successfully for studying genetic disorders most often through specific gene knockdown or deletion using homologous recombination [5] or lentiviral transduction [6]. Furthermore, in the case of some potentially fatal of untreatable conditions, hESCs have also been derived from preimplantation embryos genetically diagnosed as defective by single blastomere sampling during Preimplantation Genetic Diagnosis (PGD). Disorders that have been studied using PGD-hESC include a number of severe congenital disorders such as fragile X syndrome [7], Turner syndrome [8], and trisomy 21 [9]. However, hESC lines to investigate multifactorial and complex diseases may not be available through PGD, because they may not be considered sufficiently severe [10], as is the case for many cardiac diseases. Thus, even though hESCs are useful when there is pre-existing knowledge on the specific mutations causing the disease and the mutations can be introduced into an otherwise healthy line, hiPSCs are preferred where the entire genetic background is relevant.

The use of patient somatic cells to derive hiPSCs is also preferable in some countries, since it circumvents ethical issues that surround the destruction of human embryos for research purposes. The advent of hiPSCs has also superseded efforts to derive cloned embryos by somatic cell nuclear transfer and isolate individual hESC lines from them [2, 11, 12]. Many methods have now been described that allow somatic cell reprogramming [13]. The first and still among the most efficient methods described overexpress the reprogramming factors c-MYC, SOX2, KLF4, and OCT3/4 after retroviral or lentiviral transduction of dermal fibroblasts. This results in the integration of reprogramming genes into the genome and subsequent reactivation of the endogenous counterparts [11, 14]. Alternative non-integrating reprogramming methods are now more widely used and include the use of Sendai viruses [15], plasmids [16], and modified RNA [17]. Small molecules have also been used but have relatively lower efficiencies [18]. Somatic cell sources currently used for reprogramming not only include the original dermal fibroblasts isolated from skin biopsies, but also blood cells [19], keratinocytes from plucked hair [20], and exfoliated renal tubular epithelial cells obtained from urine [21]. Many patient-specific lines have been described that are suitable for cardiovascular disease modeling and are proving of particular value for studying disorders of unknown or complex genetic origin, as will be discussed in this review.

## Differentiation into cardiomyocytes

In vitro differentiation of hPSCs into cardiomyocytes mimics the sequential stages of embryonic cardiac development [22]. In the vertebrate embryo, the heart is one of the first organs to develop; after gastrulation, anterior migrating mesodermal cells intercalate between the ectoderm and the endoderm germ layers in the primitive streak to start generating the heart [23, 24]. Cardiac progenitor cells derive from two small tracts of epiblast cells of the developing primitive streak and take residence in the lateral plate mesoderm [25]. Signals from the surrounding tissues, such as growth factors of the WNT, BMP, and TGF-β families, are critical to promote the specification of myocardial fate. Accordingly, many of the successful protocols developed to induce cardiomyogenesis in hPSCs are based on activating and inhibiting these signaling pathways. As an example, stimulation of extraembryonic ectoderm via BMP signaling (by BMP4) and posterior primitive streak via WNT signaling (by CHIR99201) during the first 24 h of differentiation promotes the exit from self-renewal and the induction of cardiac mesoderm [26]. Moreover, inhibitors of WNT signaling, such as IWR-1, IWP-3, and XAV939, have been shown to induce cardiogenesis when added after mesoderm formation [27–29], while SB-431542, an inhibitor of the TGF-β pathway, promotes cardiogenesis when its addition occurs after mesoderm specification [30]. Current methods for cardiac differentiation of hPSCs rely on three different approaches that are summarized in Table 1, embryoid body formation, co-cultures, and monolayer culture [22].

**Table 1:** Methods for differentiating hPSCs into cardiomyocytes (modified from [34])

Differentiation	Culture conditions	Limits	Efficiency^a^ (%)	References
EBs	Serum-based media	Low efficiency Serum media	5–15	[3]
	RPMI + B27 supplement ActivinA + BMP4	Medium efficiency Batch-to-batch variability of growth factors Chemically undefined “B27”	60	[232]
	Bioreactor suspension culture RPMI + B27 supplement Small molecules	Chemical undefined “B27”	90	[233]
Inductive co-culture	Serum-based media Feeder layer Mouse END-2 cells	Low efficiency Serum media Requirement for mouse feeder cells	35	[22]
Monolayer culture	RPMI + B27 supplement ActivinA + BMP4	Low efficiency Batch-to-batch variability of growth factors Chemically undefined “B27”	35	[234]
	RPMI + B27 supplement Matrigel Sandwich ActivinA + BMP4	Batch-to-batch variability of Matrigel and growth factors Chemically undefined “B27”	90	[235]
	RPMI + B27 supplement Small molecules	Chemically undefined “B27”	90	[236]
	RPMI + human albumin l-ascorbic acid 2-phosphate Small molecules		85	[32]
	Na^+^ lactate		95	
	ActivinA + BMP4	Medium efficiency Batch-to-batch variability of growth factors	50	[237]

^a^Efficiency was calculated from flow cytometry data as the number of cells positive for cardiac troponin T (cTnT), MLC-2α, and MLC-2v, by immunostaining for MHC-β or by determining the percentage of EBs containing contracting areas

Functional cardiomyocytes can be generated from hPSCs as three-dimensional spheroid-like aggregates termed embryoid bodies (EBs), referring to their similarity with the early post-implantation embryos. Protocols to form EBs were originally developed using fetal bovine serum supplemented culture medium, but a variety of serum-free, defined media formulations are now available. Methods to form EBs from hPSCs range from an enzymatic partial dissociation of hPSC colonies, and to precise control of cell number and size by forced aggregation in microwells, to microwells in which hPSC colonies are first expanded to a defined size, to micropatterned substrates [22].

Alternatively, the early studies also used inductive co-culture of mechanically passaged hESCs with visceral endodermal-like END2 cells derived from mouse P19 embryonal carcinoma cells [31]. Notably, visceral endoderm plays a key role in the induction of cardiogenic precursor cells in development.

For ease of use though, monolayer differentiation protocols have been preferred. Benefits compared to the EB and co-culture systems include higher efficiencies and easy monitoring of outcome. Refinements over the last decade now support the generation of differentiated cell populations containing 85% cardiomyocytes; multiple methods have been described in which cardiomyocytes can be enriched to 95% using, for example, selection in sodium (Na^+^) lactate containing medium [32–34] or on the basis of cell surface markers like SIRPA and VCAM1 [28, 35].

Cardiomyocytes derived under all these culture conditions beat spontaneously, express sarcomeric proteins and ion channels, and exhibit cardiac-type action potentials (APs) and calcium (Ca^2+^) transients. Furthermore, they show similar functional properties to the cardiomyocytes in the developing heart, such as comparable dose-dependent response to cardiac drugs in terms of beating frequency and contractility, β-adrenoreceptor responses, action potential (AP) morphologies, and excitation–contraction coupling mechanisms [36]. Although opportunities still remain for improvement of reproducibility in cardiac differentiation between individual hPSC lines, reduction in the cost of reagents and in batch-to-batch variability, and of the yield and purity of required cardiomyocyte types, several protocols now support robust cardiac differentiation and some of these are available commercially as kits.

## Characterization of cardiomyocyte phenotype

The use of hPSC-CMs as a platform to model cardiovascular disorders requires their rigorous molecular and functional characterization. To maximize their potential applications in cardiovascular medicine, a qualitative comparison with adult (or fetal) primary human cardiomyocytes is advisable. Parameters used to characterize the cardiomyocyte phenotype are listed in Table 2 and include size and morphology, sarcomere structure, electrophysiological properties, Ca^2+^ handling and contractile force, responses to β-adrenergic stimulation, mitochondrial function and metabolic profile, and conduction velocity.

**Table 2:** Key features used to characterize the human cardiomyocyte phenotype

Features	Measured parameters	Human adult cardiomyocyte
Size and morphology	Shape (rod, round) Size (μm) Cell capacitance (pF)	Elongated Rod shaped ~65% mononucleated
Sarcomeres	Alignment Organization (Z lines, H zone, I bands, A bands) Molecular composition (MYH7:MYH6, MYL2:MYL7, TNNI1:TNNI3)	Organized and aligned MYH7 predominant isoform in the ventricle MYL7 predominant isoform in the atrium
Electrophysiological properties	AP (APA, RMP, *V*_max_, APD) Ion current densities and gating properties (*I*_Na_, *I*_CaL_, *I*_CaT_, *I*_to_, *I*_Kur_, *I*_Kr_, *I*_Ks_, *I*_K1_, *I*_K,Ach_, *I*_K,ATP_, *I*_f_)	Typical atrial, ventricular, pacemaker, and Purkinje AP shapes [238, 239] Distinct ion current densities and function in atrial, ventricular, pacemaker, and Purkinje cardiomyocytes [238, 239]
Ca^2+^ handling and contractile force	Ca^2+^ transients Force of contraction Ca^2+^ sparks and Ca^2+^ waves	Efficient Ca^2+^ transient induction by Ca^2+^ influx through L-type Ca^2+^ channels (Ca^2+^-induced Ca^2+^-release) [52] Force of contraction: 10–50 mN/mm^2^ (ventricular myocytes) [240] Positive force-frequency relationship (Bowditch phenomenon) [241] Low rate of spontaneous Ca^2+^ release
Response to β-adrenergic stimulation (cascade of events)	Chronotropic effect Inotropic effect Lusitropic effect	Positive chronotropic, inotropic and lusitropic effects
Mitochondrial function and metabolic profile	Oxygen consumption Glycolysis and ATP measurements Mitochondrial membrane potential Mitochondrial [Ca^2+^] Mitochondrial [Na^+^] Redox state Intramitochondrial pH ROS generation	Mitochondria occupies one-third of the total volume of CMs ATP production occurs mainly through oxidative metabolism (predominantly fatty acids)
Conduction velocity	Conduction velocity maps Expression level of ion channels and gap junction proteins Localization, density, and composition of gap junction proteins	Generation of the electrical signal through Na^+^ channels and propagation through gap junctions Localization of gap junction proteins at cell borders

### Size and morphology

In the adult heart, cardiomyocytes are elongated and rod shaped, and ~65% of them are mononucleated and this percentage does not change significantly throughout life [37, 38]. Furthermore, adult cardiomyocytes align longitudinally in the heart and are connected by intercalated discs that facilitate the electrical conduction and muscle contraction [39]. To date, despite the high differentiation efficiencies now achievable, hPSC-CMs remain small in size and round in shape [40] suggesting an immature or fetal phenotype. Several strategies have been used to mature hPSC-CMs. These include prolonged time in culture (>50 days), where hPSC-CMs become more elongated and less rounded [40] and advanced engineering approaches such as 3D platforms, either as “biowires”, or engineered heart tissues (EHTs), which allows the generation of hiPSC-CMs with improved ultrastructural and electrophysiological properties [41, 42]. Examples of improved ultrastructural properties included cardiomyocyte anisotropy with Z bands frequently visible and aligned, pronounced presence of H zones and I bands, and scattered presence of T-tubule-like structures [41, 42]. These methods as well as other maturation strategies are summarized in the “Conclusions” section of this review.

### Sarcomere structure

Human adult cardiomyocytes are characterized by organized and aligned sarcomeres [38], the smallest contractile units of striated muscles. Sarcomeres are composed of contractile proteins, including actin and myosin, which generate the force of contraction, and thin filament proteins, which calibrate the force generated by contractile proteins. In the adult ventricle, the β isoform of the protein Myosin Heavy Chain (MHC-β), encoded by the gene *MYH7*, is predominant compared to the atrial α isoform MHC-α, encoded by *MYH6* [43]; in addition, the isoform Myosin Light Chain 2v (MLC-2v), encoded by the gene *MYL2*, is predominant compared to the MLC-2α, encoded by *MYL7*, which is instead the primary human atrial isoform. Similarly, a genetic switch between the troponin I fetal (*TNNI1*) and adult isoforms (*TNNI3*) in the human heart characterizes the transition from fetal to post-natal development [44].

Sarcomeres in hPSC-CMs are less organized than in adult cardiomyocytes, and MHC-α and MLC-2α are generally highly expressed, while MHC-β and MLC-2v display relatively low level of expression [45]. In addition, the TNNI1:TNNI3 protein isoform ratio reflects a fetal stage, even after long-term culture [46]. This is partly due to hPSC-CMs being more similar to fetal cardiomyocytes but also to the heterogeneous nature of the hPSC-CMs population, which consists on a mixture of ventricular-, atrial-, and nodal-like cells. Recent engineering approaches have attempted to improve sarcomere organization and myofibril alignment in hPSC-CMs, to allow study of their structural and contractile properties, such as actin–myosin cross-bridge cycling, myofibril tension, and kinetics of activation and relaxation. Examples include the work of Salick and colleagues in which hESC-CMs were seeded onto controlled two-dimensional micropatterned rectangles made with high-resolution photolithography and microcontact printing [47], and the work of Pioner and colleagues in which hiPSC-CMs were seeded on nanogrooved surfaces and cultured long term (80–100 days) [48]. Importantly, the latter study demonstrated that myofibril tension and kinetics were similar between long-term cultures of hiPSC-CMs and second trimester human fetal ventricular cardiomyocytes. Importantly, the fetal sarcomeric properties of hPSC-CMs may represent an obstacle to faithfully recapitulating cardiomyopathy-associated phenotypes that are linked to sarcomere protein mutations. For example, the force of contraction was decreased in hiPSC-CMs with MYBPC3 mutations compared with wild-type cells, while hypertrophic cardiomyopathy (HCM) due to sarcomeric mutations is usually associated with hypercontractility [49, 50].

### Electrophysiological properties

Electrophysiological properties of adult cardiomyocytes can be described by their AP profile, which is widely considered specific for each cardiomyocyte subtype (atrial, ventricular, pacemaker, and Purkinje). However, independent of subtype, AP always starts with a rapid influx of Na^+^ as a rapid depolarizing current (*I*_Na_), termed “AP upstroke” (phase 0). Afterwards, phase 1 of the AP is characterized by a transient repolarizing current (*I*_to1_) of efflux of potassium (K^+^), followed by the inward Ca^2+^ current (*I*_CaL_) through the L-type depolarization-activated Ca^2+^ channels, which is called the plateau phase of the AP (phase 2). Next, two K^+^ currents (*I*_ks_ and *I*_kr_) drive the repolarizing phase 3 of the AP. Hence, in adult atrial and ventricular cardiomyocytes, the presence of a rectifying K^+^ current (*I*_k1_) stabilizes the resting membrane potential (RMP) at −85 mV; this is termed phase 4 of the AP.

hPSC-CMs are more depolarized compared to adult cardiomyocytes: RMP is less negative (−50/−60 mV), Na^+^ channels are fewer, and phase 0 of the AP is slow. In addition, hPSC-CMs exhibit spontaneous contractile activity, due to the absence or very low expression of *I*_k1_, and the presence of a funny current (*I*_f_), which is a pacemaker Na^+^/K^+^ hyperpolarizing current [51, 52].

Despite the differences with adult cardiomyocytes (reviewed in [53] and [54]), hPSC-CMs offer the opportunity to study some developmental- and disease-relevant cardiac properties. As an example, arrhythmogenic diseases of the heart have successfully been recapitulated using patient hiPSC-CMs, displaying significant AP changes, such as AP prolongation in the long-QT syndrome [55]. In addition, in 2013, the US Food and Drug Administration (FDA) chose hiPSC-CMs as cell type of choice for testing cardiac effects of novel compounds [51].

### Ca^2+^ handling and contractile force (excitation–contraction coupling)

The process termed “excitation–contraction coupling” (ECC) consists of the repeated contraction and relaxation of the chambers of the heart, in which Ca^2+^ is, perhaps, the most important ion involved. Ca^2+^ that enters the cell during the plateau phase of the AP enhances Ca^2+^ release from the sarcoplasmic reticulum (SR) through ryanodine receptor-2 (RYR2) channels. This causes an increase in intracellular Ca^2+^, which binds to the myofilament protein troponin C, activating the mechanism of the contraction. For relaxation, Ca^2+^ instead dissociates from troponin C and leaves the cytosol through four different systems: SR Ca^2+^-ATPase (SERCA2a); sarcolemmal Na^+^/Ca^2+^ exchanger (NCX); sarcolemmal Ca^2+^-ATPase; and mitochondrial Ca^2+^ uniport [52]. T-tubules are invaginations in the cell membrane located where L-type Ca^2+^ channels and RYR2 channels are close to each other and represent one of the most important components of the Ca^2+^ handling system, contributing to ECC [56]. To date, although hPSC-CMs express NCX at comparable levels of adult cardiomyocytes [57], the SR is still poorly developed and T-tubules have rarely been described. Consequently, Ca^2+^ handling kinetics as well as ECC are overall slow in hPSC-CMs [58].

### Responses to β-adrenergic stimulation

Sympathetic stimulation of the heart through β-adrenergic receptor agonists, such as epinephrine, activates a membrane stimulatory GTP-binding protein, which stimulates adenylyl cyclase to produce cyclic adenosine monophosphate (cAMP), which, in turn, leads to the subsequent activation of Protein Kinase A (PKA), therefore, potentiating the cardiac Ca^2+^ transients. In response to β-adrenergic stimulation, adult cardiomyocytes display positive chronotropic (increase in beating frequency), positive inotropic (increase in contractility), and positive lusitropic (acceleration of relaxation) effects [52]. Although hPSC-CMs as well as fetal cardiomyocytes do exhibit chronotropic responses to β-adrenergic stimulation [59, 60], they do not show an increase in contraction or acceleration in the relaxation period [61], unless when incorporated in human EHTs as shown by Mannhardt [42]. These considerations need to be taken into account when hPSC-CMs are used for testing the efficiency of β-adrenergic drugs on the cardiovascular system.

### Mitochondrial function and metabolic profile

Due to its incessant contraction, the heart has an extremely high energy demand compared to other tissues of the human body [52]. Mitochondrial biogenesis increases over time during heart development, so that in adult cardiomyocytes, one-third of the cell volume is, indeed, occupied by mitochondria [62]. Due to this change, during development, glucose and lactate represent the predominant substrates for the majority of ATP production in fetal cardiomyocytes, while adult cardiomyocytes mainly use fatty acids [63, 64]. Although hPSC-CMs still display an immature phenotype, they also use fatty acids for the majority of ATP production and mitochondrial density increases over time, recapitulating to a certain extent the development of the human heart [51, 65]. For this reason, hPSC-CMs have successfully been used to recapitulate and study the key aspects of mitochondrial and metabolic diseases in humans, as Drawnel and colleagues have recently showed by modeling diabetic cardiomyopathy and phenotypically screening drugs for a complication of type 2 diabetes [66].

## Conduction velocity

While the parameters above can be evaluated in single cells, the conduction velocity can only be measured in monolayer cultures. Major factors contribute to determine the conduction velocity of cardiomyocytes: propagation of the electrical signal through Na^+^ channels [67]; localization of Na^+^ channels and gap junction proteins [68]; localization, density, and composition of gap junction proteins [69]; and cell size [70]. Although the composition of gap junction proteins is similar in hPSC-CMs and adult cardiomyocytes, Na^+^ channels and gap junctions need to be distributed at the edges of two adjacent cells (adult cardiomyocytes) [71], rather than all around the cell circumference (fetal and hPSC-CMs). This, together with a reduced availability of Na^+^ channels due to a hyperpolarized RMP and cell size, contributes to the slow conduction velocity observed in hPSC-CMs [51]. Of note though, several groups have addressed this issue by repolarizing the RMP through overexpression or electronic enhancement of *I*_K1_ as a robust method to obtain more physiological electrical behaviour, including increased Na^+^ channel availability and improved Ca^2+^ transients profile [72–75]. Importantly, *I*_K1_-enhanced hiPSC-CMs displayed a stable RMP in the absence of spontaneous beating activity, allowing more accurate quantitative analysis of AP in comparing healthy and diseased myocytes [72–75]. In addition, increased cell size, membrane capacitance, and DNA synthesis were also observed [73].

## Existing hiPSC models of cardiovascular and non-cardiovascular diseases with cardiac traits

**Fig. 1:**
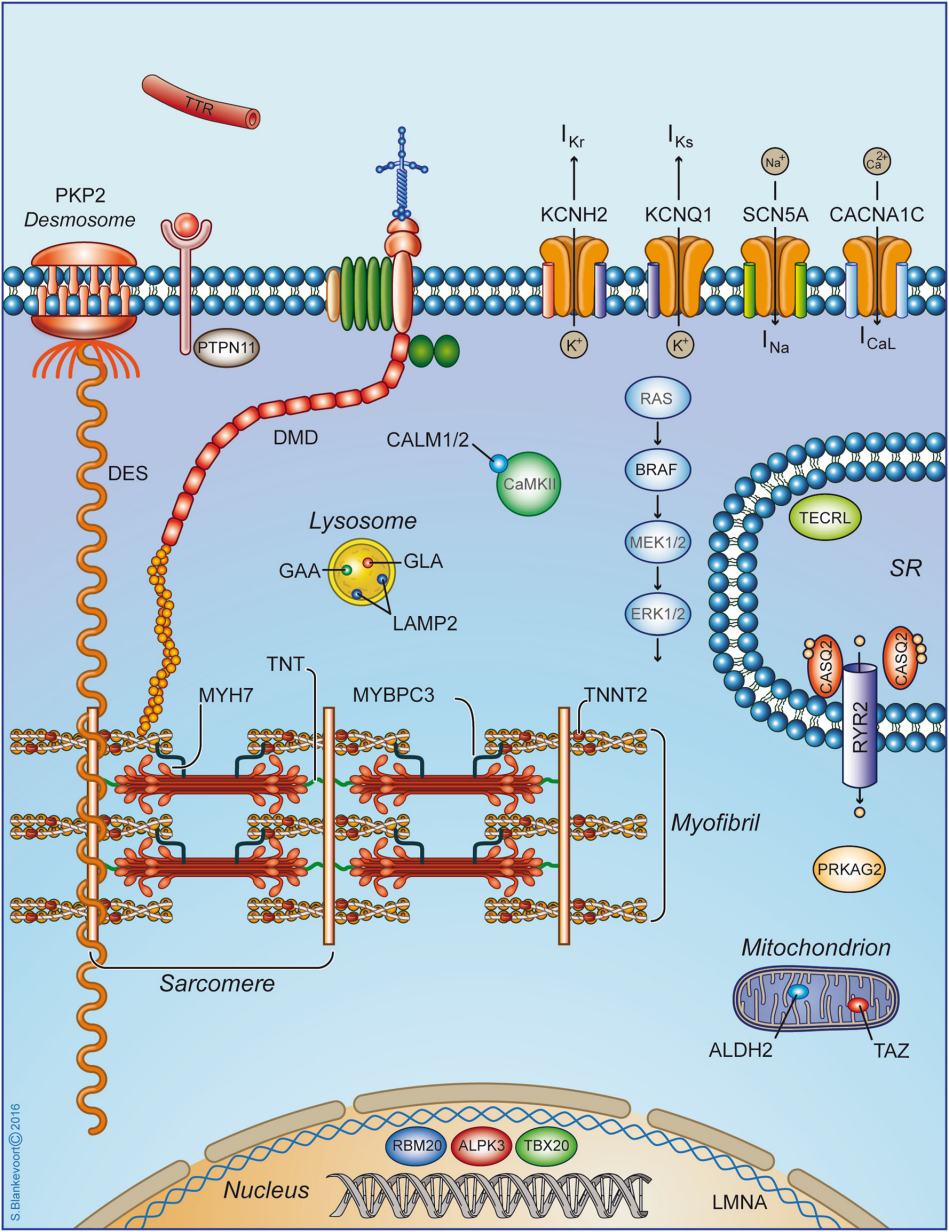
Schematic representation of cardiomyocyte structure and relevant cellular and molecular components that are mutated in cardiac diseases. This schematic shows the cardiac proteins encoded by mutated genes for which hiPSCs have been generated and reviewed here. Disease genes of interest, which are also listed in Table 3, are located in different compartments of the cardiomyocyte, such as the extracellular matrix, sarcoplasmic reticulum (SR), cytoskeleton, sarcomere, desmosome, lysosome, mitochondrion, and the nucleus

To date, hiPSC-CMs have successfully been used not only to recapitulate, but also to better understand and elucidate the disease-relevant cellular and molecular pathological mechanisms of several cardiovascular diseases. They remain one of the few opportunities to study the heart against a background of human gene expression. Below, as well as in Fig. 1 and Table 3, we list most of the hiPSC cardiac models to date and provide specific examples.

**Table 3:** Existing hiPSC models of cardiovascular diseases and disorders with cardiac traits

Disease	Gene	Mutation	References
Arrhythmias and channelopathies			
LQT1	KCNQ1	R190Q	[84]
LQT1	KCNQ1	P631 fs/33	[87]
LQT1	KCNQ1	Ex7Del	[88]
LQT1/JLNS	KCNQ1	R594Q	[111]
		E160fs+138X	
LQT1/LQT2	KCNQ1	G269S	[85]
	KCNQ1	G345E	
	KCNQ1	R190Q	
	KCNH2	A614V	
LQT2	KCNH2	G1681A	[90, 91]
LQT2	KCNH2	R176W	[93]
LQT2	KCNH2	A561V	[95]
LQT2	KCNH2	N996I	[94]
LQT2	KCNH2	A614V	[92]
LQT2	KCNH2	A561P	[97]
LQT2/LQT3	KCNH2	A422T	[96]
	SCN5A	N406K	
LQT2	TBX20	R311C	[98]
LQT3	SCN5A	V1763M	[100]
LQT3	SCN5A	V240M	[102]
		R535Q	
LQT3	SCN5A	F1473C	[101]
	KCNH2	K897T	
LQT3	SCN5A	R1644H	[103]
LQT8/TS	CACNA1C	G1216A	[107]
LQT14	CALM1	F142L	[75]
LQT15	CALM2	D130G	[110]
BrS/LQT3	SCN5A	1795insD	[74, 118, 120, 242]
BrS/LQT3	SCN5A	E1784K	[121]
BrS	SCN5A	R620H/R811H	[122]
		4189delT	
CPVT	RYR2	M4109R	[243]
CPVT	RYR2	F2483I	[126, 244]
CPVT	RYR2	P2328S	[245]
CPVT	RYR2	S406L	[127]
CPVT	RYR2	P2328S EX3del T2538R L4115F Q4201R V4653F	[128]
CPVT	RYR2	L3741P	[130]
CPVT	RYR2	I4587V	[131]
CPVT	RYR2	E2311D	[129]
CPVT	CASQ2	G112+5X	[132]
CPVT/LQTS	TECRL	SRD5A2L2	[133]
		c.331+1G>A	
Cardiomyopathies			
BTHS	TAZ	517delG	[137]
BTHS	TAZ	Gly197Val	[136]
		EX2Del	
		Arg57Leu	
Leopard	PTPN11	T468M	[140]
ARVC	PKP2	Gly828Gly	[146]
		R672fsX683	
ARVC	PKP2	L614P	[144]
ARVC	PKP2	A324fs335X	[145]
ARVC	SCN5A	R1898H	[147]
DCM	TNNT2	R173W	[152, 153]
DCM	LMNA	R225X	[151]
DCM	TTN	W976R	[156]
		A22352fs	
		P2258fs	
DCM	DES	A285V	[154]
DCM	RBM20	R636S	[157]
HCM	MYBPC3	C2373dipG	[50]
HCM	MYH7	Arg663His	[159]
HCM	BRAF	T599R	[163]
HCM	BRAF	T599R	[164]
		Q257R	
DCM/HCM	ALPK3	W1264X	[166]
HCM	PRKAG2	N488I	[168]
		R531Q	
LQT1	KCNQ1	G269S	[86]
HCM	MYH7	R663H	
DCM	TNNT2	R173W	
HCM	MYH7	R442G	[160]
HCM	MYBPC3	Arg91Cys	[161]
		N/A	
		Gly999/Gln1004del	
HLHS	N/A	N/A	[169]
IHD/CAD	ALDH2	ALDH2*2	[184]
Cardiometabolic diseases			
PD	GAA	Ex18Del	[190]
		1441delT/TRP746TER	
PD	GAA	Arg266Cys/M439K	[194]
PD	GAA	D645E/D645E	[189]
		D645E/2040-1G	
PD	GAA	Ex18del	[193]
Danon	LAMP2	129-130 insAT	[198]
		IVS-1 c.64+1 G>A	
Fabry	GLA	W162X	[201]
Fabry	GLA	W162X/R220X	[202]
Fabry	GLA	IVS4+919 G>A	[203, 204]
Diabetic cardiomyopathy	N/A	N/A	[66]
Non-cardiovascular diseases with cardiac traits			
DMD	DMD	Ex50Del	[212]
DMD	DMD	Ex45-52del	[213]
ATTR	TTR	L55P	[215]

A search for original articles published up to February 2017 was performed using PubMed Advanced Search Builder using the following criteria: (i) (human-induced pluripotent stem cells) AND (cardiac disease model) NOT review; (ii) (human-induced pluripotent stem cells) AND (cardiomyocytes) NOT review; (iii) (human-induced pluripotent stem cells) AND (cardiomyocytes) AND (mechanistic insight) NOT review. References on cardiac regeneration were manually excluded. References from some of the most comprehensive reviews of the field were screened and manually added when not present in the above-mentioned search. Limitation of this review relates to selection bias

### Arrhythmias and channelopathies

#### Familial long-QT syndrome

Long-QT syndrome (LQTS) is a potentially life-threatening arrhythmia characterized by a prolongation in the ventricular repolarization component (QT interval) of the electrocardiogram (ECG) [76]. Patients affected by LQTS experience polymorphic ventricular tachycardia with a characteristic shape of the ECG also termed “Torsades de Pointes”, syncope, and sudden cardiac death. LQTS includes hereditary variants: the autosomal-dominant form or Romano–Ward syndrome and the recessive form or Jervell and Lange-Nielsen syndrome (JLNS) [77–80]. LQTS is associated with more than 500 mutations in 16 different genes encoding cardiac ion channel proteins and their auxiliary subunits or modulating proteins, and displays a wide range of phenotypes even within members of the same family [81, 82].

#### *LQT1*

LQT1 patients harbor mutations in the *KCNQ1* gene, which encodes the K^+^ channel K_v_7.1 mediating the repolarizing current *I*_ks_ of the AP [83]. To date, several LQT1 hiPSC lines have been generated and characterized from patients carrying distinct mutations in the *KCNQ1* gene, such as R190Q [84, 85], G269S and G345E [85, 86], P631fs/33 [87], and a novel heterozygous exon 7 deletion (ex7Del) [88].

In 2010, Moretti and colleagues used retroviral vectors to generate patient-specific hiPSCs from members of a family affected by the autosomal-dominant missense mutation R190Q in the *KCNQ1* gene and differentiated the patient-derived cells into functional cardiomyocytes that recapitulated in vitro electrophysiological features of the LQT1 disease phenotype and the therapeutic approach of β-blockade [84]. In the same study, hiPSC-CMs helped demonstration of a dominant negative trafficking defect of the mutated channel. Similarly, Egashira et al. identified the same molecular mechanism as being responsible of an LQT1 phenotype in P631fs/33-KCNQ1 mutated hiPSC-CMs [87]. In another study, Liang and colleagues generated a library of hiPSC-CMs from healthy individuals and patients with different hereditary cardiac disorders, including LQT1, for recapitulating and predicting drug-induced arrhythmia. Interestingly, these cells displayed a broad spectrum of cardiotoxicity effects suggesting that disease-specific hiPSC-CMs may accurately predict adverse drug-induced cardiotoxicity [86]. Furthermore, in 2014, Wang et al. generated hiPSCs by overexpressing ion channel genes with dominant negative mutations causing LQT1 (G269S, G345E, and R190Q). To achieve stable transgene expression, these genes were integrated into the AAVS1 safe harbor locus using the Zinc Finger Nuclease technology. Next, transgene cells and isogenic unedited controls were differentiated into cardiomyocytes and recapitulated the LQT1 disease phenotype showing a prolongation in the AP duration (APD) [85].

#### *LQT2*

LQT2 patients carry mutations in the *KCNH2* gene, also termed human ether-a-go-go related gene (*hERG*), which encodes the K^+^ channel mediating the repolarizing current *I*_kr_ of the AP [89]. A panel of LQT2-diseased hiPSCs carrying the following *hERG* mutations has been generated and characterized: G1681A [90, 91], A614V [85, 92], R176W [93], N996I [94], A561V [95], A422T [96], and A561P [97].

By performing multi-electrode array, patch-clamp electrophysiology, and drug testing, Matsa et al. demonstrated that hiPSC-CMs from two patients carrying the G1681A *KCNH2* mutation showed prolonged APs but displayed different drug-induced sensitivity [90, 91]. Two independent laboratories applied similar strategies for modeling LQT2 by generating hiPSCs from patients carrying the missense A614V [92] and R176W [93] mutations on the hERG channel. However, despite the novelty of using patient hiPSC-CMs for modeling LQT2, these studies were performed under genetically non-defined conditions and, therefore, genetic background variations were not taken into account. To address this limitation, we modeled LQT2 syndrome by generating hiPSCs from a patient carrying the N996I hERG missense mutation and corrected the mutation by homologous recombination. Next, we introduced the same mutation in hESCs, generating two genetically distinct isogenic pairs of LQTS and control lines [94]. This approach allowed the electrophysiological changes to be attributed to the specific mutation. In another study, hiPSCs were derived using a virus-free method from patients with the A561V missense mutation in the *KCNH2* gene and they differentiated them into beating cardiomyocytes. Notably, this study provided an approach to rescue the diseased LQT2 phenotype correcting hERG trafficking defects with the pharmacological agent ALLN, demonstrating with patient-specific hiPSC-CMs that re-trafficking of the mutated channels might represent an alternative approach for some *KCNH2* mutations [95].

Recently, the use of hiPSC-CMs for modeling LQT2 helped revealing a key role for the transcription factor TBX20 in the regulation of *KCNH2* expression [98]. In this study, Caballero and colleagues investigated the electrophysiological effects of the R311C-TBX20 mutation, which is found in individuals affected by LQTS, in hiPSC-CMs. The authors showed that the R311C mutation specifically disables the posttranscriptional activity of TBX20 over *KCNH2*, which decreases the *I*_Kr_ and prolongs the AP, therefore, identifying TBX20 as an LQT2-modifying gene [98].

#### *LQT3*

LQT3 patients usually carry gain-of-function mutations in the *SCN5A* gene, which encodes the Na^+^ channel Na_V_1.5 mediating the fast depolarizing current *I*_Na_ during AP [99]. To date, several SCN5A mutations have been modeled with patient-specific hiPSC-CMs: V1763M [100], F1473C [101], V240M and R535Q [102], and R1644H [103].

In 2013, Ma and colleagues derived hiPSC-CMs from an LQT3 patient harboring a V1763M-SCN5A mutation and recapitulated the biophysical abnormalities (prolonged APD, increased tetrodotoxin (TTX)-sensitive late or persistent Na^+^ current, positive shift of steady-state inactivation, and faster recovery from inactivation) of the disease. In this study, the hiPSC line was generated from dermal fibroblasts of the patient and control-hiPSC-CMs were derived from the healthy sister of the patient [100]. However, LQTS may occur in families whose members are affected by multiple mutations and complex genetics. Such disease phenotypes are difficult to recapitulate in vitro; moreover, the development of patient-specific clinical regimens remains challenging. To address these limitations, hiPSC-CMs have been generated from family members with complex genetics, such as reported by Terrenoire et al. [101]. In this study, hiPSCs were derived from an LQTS patient harboring the F1473C SCN5A mutation and the K897T KCNH2 polymorphism. Notably, analysis of the biophysics and molecular pharmacology of ion channels expressed in cardiomyocytes differentiated from these cells displayed a primary LQT3 Na^+^ channel defect responsible for the patient’s arrhythmias, which was not influenced by the *KCNH2* polymorphism. In a similar manner, Fatima et al. reported the generation of hiPSCs from two LQT3 patients carrying two distinct mutations in SCN5A (V240M and R535Q), which resulted in defective biophysical properties of Nav1.5 [102]. Furthermore, in a large family affected by congenital LQT3 syndrome, 15 out of the 23 available individuals were identified as heterozygous carriers of the missense mutation R1644H in *SCN5A*. Of note, Malan and colleagues obtained skin biopsies from one member of this family affected by LQT3, as well as from one healthy control individual of the same family [103]. Of particular interest, after addition of mexiletine, a Na^+^ channel inhibitor commonly used in LQT3 therapy, a shortening in the APD was noticed in LQT3 hiPSC-CMs, which successfully rescued the disease phenotype of the patient.

#### *LQT8/Timothy syndrome* (*TS*)

LQT8, also known as Timothy syndrome (TS), is a complex multi-system disorder characterized by QT prolongation, webbed fingers and toes, flattened nasal bridge, low-set ears, small upper jaw, thin upper lip, and typical autism traits [104, 105]. TS patients carry mutations in the *CACNA1C* gene, which encodes the Ca^2+^ channel Ca_V_1.2, the main L-type Ca^2+^ channel in the mammalian heart responsible for the plateau phase of the AP and essential for ECC [106]. Yazawa and colleagues successfully modeled the cardiac phenotype of TS including irregular contraction and electrical activity, and abnormal Ca^2+^ handling by generating hiPSC from a patient harboring a G1216A missense mutation in *CACNA1C* [107]. Of particular interest, the small molecule roscovitine proved successful in restoring normal electrical and Ca^2+^ properties.

#### *LQT14*

Patients carrying mutations in one of the three genes encoding calmodulin (CaM, a multifunctional intermediate Ca^2+^-binding messenger protein essential for the functionality of the heart, immune system, and brain) manifest cardiac arrhythmias associated with severe LQTS, as well as catecholaminergic polymorphic ventricular tachycardia and idiopathic ventricular fibrillation [108–110]. Mutations in the *CALM1* gene, encoding CaM, are associated with type 14 LQTS (LQT14). In this regard, Rocchetti and colleagues recently investigated the unclear arrhythmogenic effect of the heterozygous F142L mutation in *CALM1* by studying patient-specific hiPSC-CMs electrophysiology with addition of stimulated *I*_k1_ by Dynamic-Clamp [75]. Mutated hiPSC-CMs displayed loss of *I*_caL_ inactivation and abnormal APD, whilst *I*_ks_ and *I*_NaL_ remained unaltered. *I*_caL_ blockage rescued the disease phenotype. Importantly, these findings demonstrated that F142L-CaM arrhythmogenesis is caused by loss of *I*_caL_ inactivation [75].

#### *LQT15*

*CALM2* mutations are associated with type 15 LQTS (LQT15). In a recent study, Limpitikul and colleagues generated hiPSC-CMs from a patient carrying the D130G-CaM mutation within the *CALM2* gene. Notably, the patient-derived iPSC-CMs showed prolongation of the APD and disruption of Ca^2+^/CaM-dependent inactivation (CDI) of L-type Ca^2+^ channels. Importantly, allele-specific suppression of the mutated *CALM2* gene using CRISPR interference resulted in functional rescue in the hiPSC-CMs, with normalization of APD and CDI after treatment [110].

#### *JLNS*

The Jervell and Lange-Nielsen syndrome is inherited as an autosomal recessive trait and is characterized by a severe QT interval prolongation at the ECG and by deafness [78]. JLNS patients harbor homozygous or compound heterozygous mutations in *KCNQ1* or *KCNE1* genes. In one study, both patient-derived and engineered hiPSCs carrying the E160fs + 138X or the R594Q KCNQ1 mutations recapitulated the severe JLNS electrophysiological phenotype including APD prolongation and drug-induced arrhythmia susceptibility [111].

#### Brugada syndrome

Brugada syndrome (BrS) is an inheritable channelopathy characterized by a coved-type ST-segment elevation in the right precordial leads of ECG and increased risk of sudden cardiac death from ventricular fibrillation [112, 113]. Loss-of-function mutations in the *SCN5A* gene encoding the Na^+^ channel responsible for the cardiac *I*_Na_ are associated with BrS; they account for ~20% of cases [114, 115]. Genetic alterations in additional genes encoding Na^+^, K^+^, and Ca^2+^ channels or associated proteins have been linked to BrS [116]; however, ~70% of BrS patients remain genetically unsolved, suggesting that additional factors, such as copy number variations, mutations in yet-unknown genes, epigenetic factors, and post-translational modifications may contribute to this disease [117].

The 1795insD SCN5A mutation underlying both BrS and LQT3 was identified in a large Dutch family with ECG features of bradycardia and ventricular and atrial conduction slowing [118, 119]. In a study performed by Davis and colleagues, hiPSC were generated from a patient carrying the 1795insD mutation and differentiated toward cardiomyocytes that displayed the overlapped *I*_Na_ and AP properties of both BrS and LQT3 channelopathies (decrease in *I*_Na_ density, large persistent *I*_Na_, reduced upstroke velocity, and prolonged APD) [120]. Similarly, Okata et al. generated hiPSCs from a patient carrying the E1784K SCN5A mutation, which has previously been associated with the mixed phenotype of LQT3/BrS. Interestingly, electrophysiological analysis showed that LQT3/BrS-hiPSC-CMs recapitulated the phenotype of LQT3 but not BrS. Due to the fact that *SCN3B* is the predominant Na^+^ channel β-subunit in fetal hearts as well in hiPSC-CMs, while *SCN1B* is the predominant β-subunit in the adults, the knockdown of *SCN3B* in the LQT3/BrS-hiPSC-CMs successfully unmasked the phenotype of BrS. Moreover, corrected-LQT3/BrS-hiPSC-CMs exhibited the normal electrophysiological phenotype [121].

In another study of interest, Liang and colleagues generated hiPSC-CMs from two patients affected by BrS; the first patient carrying the double missense mutation (R620H and R811H) in SCN5A and the second patient carrying one base-pair deletion mutation in *SCN5A* (4189delT) [122]. Importantly, BrS iPSC-CMs successfully recapitulated features of the BrS disease, such as the reduction of inward Na^+^ current density and reduction of maximal upstroke velocity, increased triggered activity and abnormal Ca^2+^ handling [122].

However, a dysfunction in the cardiac Na^+^ channel may not always represent a prerequisite for BrS phenotype in vitro, as demonstrated by Veerman and colleagues [74]. In this study, a comparison of electrophysiological properties between hiPSC-CMs generated from three patients affected by BrS and two unrelated controls revealed no significant differences in *I*_Na_ and in upstroke velocity, therefore, indicating that the BrS phenotype here could not be recapitulated in the hiPSC model. These results led to the hypothesis that other mechanisms than ion channel defects might underlie the phenotype in these patients, such as fibrosis, decreased cardiomyocyte coupling, and environmental factors; alternatively, or in addition, immaturity of hiPSC-CMs might have hampered the detection of the disease phenotype.

#### Catecholaminergic ventricular tachycardia

Catecholaminergic polymorphic ventricular tachycardia (CPVT) is an inherited cardiac disorder characterized by ventricular tachyarrhythmia, syncope and sudden cardiac death usually induced by emotional and physical stress [105, 123]. CPVT is caused by mutations in the *RYR2* gene, which leads to the CPVT1 variant, or by mutations in the calsequestrin-2 gene (*CASQ2*), which leads to the CPVT2 variant [124]. As previously mentioned, *RYR2* encodes the principal Ca^2+^ releasing channel expressed in the membrane of the SR, while *CASQ2* encodes a high-capacity and low-affinity Ca^2+^-binding glycoprotein of the SR, both key players in ECC [125].

To date, several models of patient-specific hiPSC-CMs carrying *RYR2* mutations have been generated. Importantly, all these studies successfully demonstrated that hiPSC-CMs can recapitulate some of the Ca^2+^ handling abnormalities typical of CPVT1 and, therefore, opened new opportunities for the investigation of the disease mechanisms in vitro as well as for drug testing. As an example, Fatima and colleagues demonstrated that patient-specific hiPSC-CMs harboring the F2483I mutation in the RYR2 channel displayed arrhythmias and delayed after depolarizations (DADs) post-catecholaminergic stimulation, and higher amplitudes and longer durations of spontaneous Ca^2+^ release events at basal state when compared to healthy controls. Of note, these Ca^2+^ release events continued even after repolarization and were abolished by increasing the cytosolic concentration of cAMP with forskolin, an adrenergic stimulator that acts via production of cAMP [126]. In another study of interest, Jung and colleagues successfully restored normal Ca^2+^ spark properties and rescued the arrhythmogenic S406L RYR2 phenotype by addition of dantrolene, a drug against malignant hyperthermia. Moreover, their findings suggested that the pathogenesis of the S406L mutation is due to a defect of inter-domain interactions within the RYR2 channel [127]. The antiarrhythmic effect of dantrolene was also assessed by Penttinen and colleagues in six patients carrying various *RYR2* mutations and in their corresponding hiPSC-CM models [128]. This study showed similar patient-to-patient variation in dantrolene effects both in the patients and in the corresponding iPSC-CMs, suggesting that it may be possible to predict personalized drug-dose responses in vitro without predisposing the patient to the potentially severe side-effects of a drug [128]. In another study, Di Pasquale et al. developed a model of CPVT1 by generating hiPSCs from a patient harboring the E2311D *RYR2* mutation. Treatment of hiPSC-CMs with KN-93, a specific antiarrhythmic drug that inhibits Ca^2+^/calmodulin-dependent serine–threonine protein kinase II (CaMKII), decreased DADs, and successfully rescued the arrhythmic phenotype induced by catecholaminergic stress [129]. Interestingly, a recent study performed by Preininger and colleagues revealed the inadequacy of β-blocker treatment by nadolol in one patient affected by a novel mutation in *RYR2* that causes CPVT1 [130]. hiPSC-CMs generated from the patient showed persistent ventricular arrhythmias during β-blockade with nadolol, whereas no arrhythmias were observed during treatment with the Na^+^ channel blocker flecainide. In detail, nadolol treatment during β-adrenergic stimulation achieved negligible reduction of Ca^2+^ wave frequency and failed to rescue the Ca^2+^ spark defects in diseased hiPSC-CMs. On the other hand, flecainide reduced both frequency and amplitude of Ca^2+^ waves and restored the Ca^2+^ sparks to the baseline levels [130], closely recapitulating drug treatment in the patient. In a similar manner, Sasaki and colleagues combined electrical pacing with CPVT- and control-hiPSC-CMs to validate S107, a drug that stabilizes the closed state of the RYR2, as potential therapeutic agent for CPVT1 [131].

After proving the efficacy of Adeno-associated virus (AAV)-mediated *CASQ2* gene replacement therapy for CPVT2 in mouse models, Lodola and colleagues investigated the efficacy of this strategy in hiPSC-CMs generated from a patient carrying the homozygous G112+5XCASQ2 mutation [132]. HiPSC-CMs infection with AAV carrying the wild-type *CASQ2* gene revealed to be sufficient to restore the physiological expression of CASQ2 protein, and to observe decrease in the percentage of DADs following adrenergic stimulation as well as normalization of Ca^2+^ transient amplitude and Ca^2+^ sparks. These findings show the potential of gene therapy as curative approach in patients affected by some CPTV mutations [132].

*CPVT/LQTS—*A recent study by Devalla and colleagues was carried out on hiPSC-CMs from patients from three different families with clinical arrhythmias and high risk of sudden cardiac death [133]. Precisely, two of these patients were diagnosed with LQTS, whereas the third patient belongs to a family diagnosed with the early onset and highly malignant form of CPVT. Of note, all of them carried mutations in the gene encoding the *trans*-2,3-enoyl-CoA reductase-like protein (*TECRL* gene), whereas no mutations in the most common LQTS and CPVT genes. Analysis of intracellular Ca^2+^ dynamics, AP measurements, stimulation by noradrenaline, and treatment with the antiarrhythmic drug flecainide in the patient-specific hiPSC-CMs recapitulated the clinical phenotypes of LQTS and CPVT, showing, for the first time, that mutations in the *TECRL* gene are associated with inherited arrhythmias with clinical features of both LQTS and CPVT [133].

### Cardiomyopathies

#### Barth syndrome

Barth syndrome (BTHS) is an X-linked cardiac and skeletal mitochondrial myopathy caused by mutations of the gene *Tafazzin* (*TAZ*) [134] responsible for remodeling cardiolipin, the major phospholipid of the mitochondrial inner membrane [135]. To date, two independent studies generated BTHS hiPSCs [136, 137]. Interestingly, Wang and colleagues recapitulated the pathophysiology of BTHS cardiomyopathy by combining patient-derived hiPSCs with genome editing, modified RNAs, and “heart on a chip” technologies [137]. They demonstrated that a mutation in *TAZ* gene (517delG) is sufficient to disassemble the structure of the cardiomyocyte sarcomeres. Furthermore, they demonstrated that BTHS cardiomyopathy can be reversed by either reintroducing the wild-type *TAZ* gene, or by suppressing the level of reactive oxygen species (ROS) produced by BTHS mitochondria. In another study of interest, Dudek and colleagues studied mitochondrial oxidative phosphorylation in BTHS-hiPSC-CMs, which displayed a severe decrease in basal oxygen consumption rate and in the maximal respiratory capacity when compared to wild-type cells, leading to a dramatic increase of ROS production [136].

#### Leopard syndrome

LEOPARD is the acronym of “Lentigines, Electrocardiographic abnormalities, Ocular hypertelorism, Pulmonary valve stenosis, Abnormal genitalia, Retardation of growth, Deafness”, an autosomal-dominant disease that belongs to a class of disorders associated with RAS–mitogen-activated protein kinase signaling [138, 139]. Approximately 90% of LEOPARD syndromes are caused by missense mutations in the *PTPN11* gene, which encodes the ubiquitously expressed tyrosine phosphatase protein SHP2, although hypertrophic cardiomyopathy remains the most common abnormality in patients affected by LEOPARD syndrome [139]. Against this background, Carvajal-Vergara and colleagues generated hiPSCs from two patients with the heterozygous T468M mutation in the *PTPN11* gene and highlighted important molecular mechanisms in the signaling pathways responsible for the cardiac hypertrophic phenotype in LEOPARD syndrome, such as the increased phosphorylation of specific proteins such as MEK1 in LEOPARD-hiPSC-CMs compared to wild-type, demonstrating that RAS-MAPK signaling is perturbed in LEOPARD syndrome [140].

#### Arrhythmogenic right ventricular cardiomyopathy

Another inherited cardiac disorder that has been modeled with hPSC-CMs is the arrhythmogenic right ventricular cardiomyopathy (ARVC), characterized by the replacement of cardiomyocytes with fatty or fibrofatty tissue [141]. Approximately half of the patients affected by ARVC carry a mutation in one of the genes encoding for key components of the desmosome, the intercellular junction of cardiac muscle [142, 143]. Of note, different laboratories studied ARVC hiPSCs from patients with mutations in the *PKP2* gene, which encodes the plakophilin-2 desmosomal protein [144–146]. In these studies, fibroblasts were reprogrammed into hiPSC via retrovirus infection and cardiomyocytes were generated using 3D protocols of differentiation. Gene expression profiling, immunofluorescence staining of desmosomal proteins, transmission electron microscopy, and exposure of the cells to apidogenic stimuli allowed these scientists to successfully recapitulate the ARVC phenotype in vitro and provided mechanistic insights into the early disease pathogenesis, such as the association of ARVC phenotype with the upregulation of the pro-adipogenic transcription factor peroxisome proliferator-activated receptor-γ (PPAR-γ) [145].

Notably, it has recently been suggested an interaction between the desmosome and the Na^+^ channel protein Na_v_1.5 encoded by the *SCN5A* gene, raising the hypothesis that mutations in this Na^+^ channel complex may lead to ARVC cardiomyopathy [147]. On this note, Riele and colleagues generated hiPSC-CMs from an ARVC patient harboring the rare mutation (R1898H) in SCN5A and no desmosomal mutations. In this study, the authors demonstrated reduced Na^+^ current and Na_v_1.5/N-Cadherin clusters at junctional sites in the patient-derived hiPSC-CMs, suggesting that Na_v_1.5 may be part of a functional complex with adhesion molecules such as N-Cadherin, which reveals a non-canonical mechanism by which *SCN5A* mutations lead to ARVC cardiomyopathy [147].

#### Familial dilated cardiomyopathy

Dilated cardiomyopathy (DCM) is an inherited cardiac disorder that mostly affects the myocardium. It is characterized by left or biventricular dilatation, which is sufficient to cause global systolic impairment [148]. DCM is a genetically heterogeneous disease that can be caused by mutations in many different genes [149]. One of the key genes identified in familial DCM is *LMNA*, which encodes intermediate filament proteins of the nuclear lamina, the “lamin A/C proteins” [150]. Two different LMNA mutations, the autosomal-dominant non-sense R225X and a frame shift mutation, were investigated in a work from Siu and colleagues [151]. This study revealed that haploinsufficiency due to R225X mutation was associated with accelerated nuclear senescence and apoptosis of patient-specific hiPSC-CMs under electrical stimulation, which was attenuated by pharmacological blocking of ERK1/2 signaling pathway. Another gene associated with DCM is *TNNT2*. So far, three independent groups succeeded in showing hypertrophic signatures in hiPSC-CMs carrying the R173W TNNT2 mutation [86, 152, 153]. Furthermore, as demonstrated by Tse and colleagues, patient-specific hiPSC-CMs can be used to confirm histological and functionally suspected genetic bases for DCM [154]. In this study, using whole-exome sequencing, Tse et al. identified the novel heterozygous mutation A285V in the muscle-specific intermediate filament protein Desmin (encoded by the *DES* gene) responsible for the cytoskeletal organization between cardiomyocytes and striated muscle cells [155]. Nevertheless, the most common genetic cause for DCM consists of mutations that truncate the massive sarcomeric protein Titin (encoded by the *TNN* gene), the so-called “TTN-truncating variants” (TTNtvs), such as the W976R, A22352fs, and P2258fs mutations, studied by Hinson and colleagues [156]. Here, RNA sequencing and functional analyses were combined with cardiac engineered microtissues from healthy, mutated, and isogenic hPSC lines to demonstrate that truncations in the A-band domain of *TTN* cause DCM, whereas truncations in the I band are better tolerated, because alternative splicing excludes I-band exons from most mature *TTN* transcripts [156]. Finally, by investigating stage-specific cardiogenesis in hiPSC carrying mutations in the RNA-binding motif protein 20 gene (*RBM20*), Wyles et al. showed that in this specific case, DCM is a developmental disorder [157].

#### Familial hypertrophic cardiomyopathy

Hypertrophic cardiomyopathy (HCM) is an inherited cardiac disorder that can be caused by more than 1400 mutations in at least 11 genes encoding the thick and thin contractile myofilaments or the Z-discs of the sarcomere, leading to an abnormal thickness of the myocardial left ventricle [158]. Although the majority of individuals affected by HCM are asymptomatic or manifest mild symptoms, they are equally exposed to a high risk of progressive heart failure, arrhythmia, and sudden cardiac death [105]. However, the pathways by which sarcomeric mutations induce cardiomyocyte hypertrophy and electrophysiological abnormalities are still not completely clear [159]. Therefore, the generation of patient-specific hiPSC-CMs to model HCM may help to elucidate and, maybe, in the future, to predict the onset and the development of HCM, as demonstrated by Lan and colleagues [159]. In this study, hiPSC-CMs were generated from patients harboring the missense R663H MYH7 mutation. These cells showed enlarged cell size and contractile arrhythmia at the single-cell level. Furthermore, Ca^2+^ analysis revealed deregulation of Ca^2+^ cycling and Ca^2+^ intracellular concentration, and key mechanisms of HCM pathogenesis. Similarly, two other groups recapitulated the disease phenotype of HCM by generating hiPSC-CMs from patients carrying mutations in the *MYH7* gene [86, 160].

In two other studies, hPSC-CMs carrying a mutation in *MYBPC3,* the gene encoding the cardiac myosin-binding protein C, were generated [50, 161]. After generating hPSC-CMs from three patients with HCM, Tanaka and colleagues demonstrated that the HCM phenotype as well as the contractile variability observed in the three classes of HCM hPSC-CMs were caused by interactions between the patient’s genetic backgrounds and the cardiomyocyte hypertrophy-promoting factor endothelin-1 [161]. In another study of interest, Birket and colleagues showed that, under optimized conditions for cardiomyocyte function, which included the presence of thyroid hormone, insulin growth factor-1, and dexamethasone, single HCM hPSC-CMs showed lower contractile force when compared to controls [50].

HCM can also affect individuals with cardiofaciocutaneous syndrome (CFCS), a genetic disease characterized by abnormal RAS/MAPK signaling in multiple populations of cardiac cell progenitors [162]. In a recent study, Cashman et al. generated a 3D model of human engineered cardiac tissue, termed “hECT”, using hiPSC-CMs from patients carrying BRAF mutations and presenting with CFCS and HCM [163]. After 1 week in culture, BRAF-hECTs exhibited several structural, molecular, and functional features of hypertrophic phenotype when compared to hECTs derived from healthy individuals (larger cross-sectional area, increased expression level of the hypertrophic marker *ANP*, increased expression of the hypertrophic marker *BNP,* and the Ca^2+^ regulatory marker *SERCA2a*, as well as greater developed force, shorter twitch duration, and higher maximum rates of contraction and relaxation). Furthermore, a model consisting on BRAF-mutated hiPSC-CMs not only recapitulated the disease phenotype of HCM, but also helped elucidating the role of RAS/MAPK signaling in HCM pathogenesis [164]. Here, Josowitz and colleagues demonstrate that activation of this pathway through TGFβ signaling leads to cardiomyocyte hypertrophy driven by both autonomous and non-autonomous cardiomyocyte defects. Importantly, these findings suggest a potential therapeutic use of TGFβ inhibitors in HCM and CFCS patients, for which no curative options exist to date [164].

Another study conducted on three unrelated families demonstrated that pediatric HCM can be caused by biallelic truncating mutations in the gene encoding the α-kinase-3 (ALPK3) [165]. Notably, several features of DMC, such as alterations in the systolic function, were also found in the same individuals, suggesting a role for the ALPK3 pathway in the pathogenesis of a mixed DCM/HCM phenotype. Subsequently, Phelan et al. derived cardiomyocytes from a consanguineous family harboring a novel biallelic truncating mutation, and from hESCs lacking ALPK3. Ultrastructural analysis, multi-electrode array, and Ca^2+^ imaging on these cells revealed disorganized sarcomere structures and intercalated discs, extended field potential duration, and increased irregular Ca^2+^ transients (arrhythmia) indicative of abnormal Ca^2+^ handling. Collectively, this study suggests that mutations in ALPK3 can cause familiar cardiomyopathy, identifying abnormal Ca^2+^ handling as a potential feature of cardiomyocytes lacking ALPK3 [166].

In addition, several missense mutations causing HCM have been observed in the gene encoding PRKAG2, one of the three regulatory subunits of the AMP-activated protein kinase (AMPK) that is highly expressed in the heart and involved in glucose handling and mitochondrial biogenesis [167]. Using hiPSC-CMs, three-dimensional cardiac microtissues, RNA sequencing, and metabolomics, Hinson and colleagues recently revealed key links between AMPK and cardiomyocyte survival and metabolism with TGFβ signaling. By demonstrating that AMPK inhibits TGFβ production and fibrosis in vivo, the authors suggest that molecules that activate AMPK may be beneficial for the treatment of fibrosis and HCM [168].

### Hypoplastic left heart syndrome

Hypoplastic left heart syndrome (HLHS) is characterized by underdevelopment of the left side of the heart which can lead to variable complications like hypoplasia or atresia of the left ventricle, ascending aorta, and aortic and mitral valves [169]. It has been suggested that HLHS may be due to a diminished blood flow through the left side of the heart [170, 171], or to the disruption of specific genetic networks required for left ventricular chamber development [172, 173]. In a study from Jiang and colleagues, dermal fibroblasts were obtained from the skin biopsy of one HLHS patient and were reprogrammed to hiPSCs [169]. Interestingly, mutated hiPSC-CMs displayed gene expression and functional differences when compared to healthy control cardiomyocytes: reduced expression of CX43 and cTnT; higher expression of *CD31* and embryonic atrial myosin essential light chain (*ALC*-*1*); higher expression of *MYH6* and decreased expression of *MYH7*; lower numbers and beating rates of contractile areas; accelerated rate of Ca^2+^ transient decay; RYR2 dysfunction; and upregulation of IP3-receptor expression. Collectively, these findings demonstrated that HLHS-disease hPSC-CMs show developmental and/or functional defects that could compromise their ability to contribute to normal cardiogenesis in vivo.

### Ischemic heart damage and coronary artery disease

A decrease of oxygen concentration in the heart tissue dramatically alters the metabolism of cardiomyocytes by producing high oxidative stress. To date, it is known that oxidative stress and ROS play a key role in Ischemic Heart Damage (IHD) and Coronary Artery Disease (CAD) pathogenesis [174]. Indeed, during Myocardial Infarction (MI), ROS cause oxidative damage such as lipid peroxidation and enhanced production of toxic aldehydes [175–177]. Moreover, the high concentration of ROS during ischemia–reperfusion triggers apoptosis and necrosis in the heart tissue [178].

Because of the more complex nature of IHD and CAD compared with cell-autonomous genetic cardiac diseases, IHD and CAD are more difficult to recapitulate in vitro with hiPSC–CMs [179]. Nevertheless, some examples are starting to emerge, suggesting that some aspects might be recapitulated and elucidated in a culture dish. Interestingly, IHD and increased risk of CAD have been linked to the single-nucleotide polymorphism E487K in the cardioprotective enzyme aldehyde dehydrogenase-2 (ALDH2*2) [180–183]. Ebert et al. generated hiPSC-CMs carrying the heterozygous ALDH2*2 allele and showed that, under ischemic conditions, these cells displayed high levels of ROS and toxic aldehydes, which led to cell cycle arrest and activation of apoptotic signaling pathways [184]. These findings highlighted the key role of ALDH2 in modulating cell survival decisions. Overall, these insights into molecular mechanisms of ALDH2*2-related ischemic damage might be useful for the development of patient-specific diagnostic methods and therapies against IHD and CAD.

### Cardiometabolic diseases

#### Pompe disease

Pompe disease (PD) is an autosomal recessive disorder caused by mutations in the gene encoding the lysosomal glycogen-degrading enzyme, acid α-glucosidase (GAA) [185, 186]. Patients affected by PD manifest reduced GAA activity, increased cytoplasmic glycogen level, mitochondrial aberrance, and progressive autophagy [187]. PD can be classified either as infantile-onset form, characterized by progressive weakness of skeletal muscle and cardiac hypertrophic cardiomyopathy, or late-onset form, and characterized by later and slower progressive weakness of skeletal muscle [188]. Importantly, the first hiPSC model of PD was generated by Huang and colleagues [189]. Since the heart is one of the most affected organs especially in the infantile-onset form of PD, Huang et al. examined whether cardiomyocytes derived from infantile PD-hiPSCs exhibited the pathophysiological features of the disease by comparing their GAA activity, glycogen content, mitochondrial function, and ultrastructural changes with healthy hiPSCs-CMs. PD-hiPSC-CMs displayed depressed GAA activity, higher glycogen content, lower oxygen consumption rate, lower extracellular acidification rate, and some but not all the ultrastructural abnormalities, such as freely dispersed glycogen [189]. Since the mechanism by which loss of GAA activity causes cardiomyopathy in the infantile-onset form of PD is not well understood, Raval and colleagues reprogrammed fibroblasts from patients affected by infantile PD and generated additional hiPSCs-CMs to gain further insight into the molecular mechanisms. Unexpectedly, they found that the lysosome-associated membrane proteins LAMP1 and LAMP2 from PD-hiPSC-CMs displayed higher electrophoretic mobility compared with healthy hiPSC-CMs. Collectively, this study suggested that PD-hiPSC-CMs produce LAMPs lacking appropriate glycosylation and that misglycosylation in these proteins may contribute to the pathophysiology of Pompe cardiomyopathy [190]. Although it has been reported that cardiovascular complications mostly affect the infantile-onset form of PD, several groups demonstrated that late-onset PD patients can also be affected, although in a less severe and frequent manner [191, 192]. To investigate this, Sato and colleagues generated late-onset PD-hiPSCs and successfully differentiated cardiomyocytes from both PD and control hiPSCs. Importantly, massive accumulation of glycogen in the lysosome of cardiomyocytes derived from PD-hiPSCs, not from control, was observed, but there were no significant differences in the structure of the cardiomyocyte fiber, such as disarray and hypertrophy. In another study of interest, Higuchi et al. compared hiPSCs generated from patients with infantile- and late-onset forms of PD [193]. Notably, ultrastructural features of these hiPSCs revealed massive accumulation of glycogen granules in the lysosomes of patients affected by infantile PD, and a few lysosomes in patients affected by the late-onset form of the disease. Collectively, these data show that cellular pathology of late-onset PS is reflected in patient-specific hiPSC-CMs [194]. Furthermore, when treated with recombinant human GAA (rhGAA), glycogen granules of infantile hiPSCs significantly decreased in a dose-dependent manner, confirming that enzyme replacement therapy improves the survival period as well as the muscle symptoms in some PD patients [195].

#### Danon disease

Danon disease is a familial cardiomyopathy characterized by impaired autophagy due to mutations in the gene encoding the lysosomal-associated membrane protein type 2 (*LAMP2*) [196, 197]. Patients affected by Danon disease display severe cardiac and skeletal muscle abnormalities resulting in heart failure and consequent sudden cardiac death [198].

Hashem and colleagues generated five independent hiPSC lines from two patients affected by Danon disease and compared them with two wild-type hiPSC lines derived from healthy unrelated individuals [198]. Importantly, all healthy and disease hiPSC-CMs expressed the cardiac-specific contractile protein α-actinin, but only Danon hiPSC-CMs lacked LAMP2 protein. Next, size, gene expression and functionality of hiPSC-CMs were examined to investigate whether they recapitulated the heart failure phenotype observed in Danon patients. Cytological analysis revealed that Danon hiPSC-CMs were significantly larger compared to healthy hiPSC-CMs, therefore, recapitulating the hypertrophy observed in the patients. Furthermore, some but not all Danon hiPSC-CMs exhibited longer Ca^2+^ decay compared to healthy controls, consistent with the decrease of systolic and diastolic function typical of heart failure [199, 200].

#### Fabry disease

Fabry disease is a rare X-linked metabolic disorder characterized by deficiency of the enzyme α-galactosidase and encoded by the *GLA* gene, causing progressive lysosomal accumulation of globotriaosylceramide (GL-3) in the kidney, heart, and other tissues throughout the body [201].

In 2013, Kawagoe and colleagues generated hiPSCs from human fibroblasts of patients affected by Fabry disease. Electron microscopic analysis indicated that Fabry-hiPSCs exhibited massive accumulation of membranous cytoplasmic body (MCB) in the lysosomes, which is typical of Fabry disease, and they could not be easily differentiated into cardiomyocytes due to the continuous damages of the intracellular architecture [201]. By contrast, in a study by Itier and colleagues, hiPSCs generated from Fabry patients were successfully differentiated toward the cardiac fate [202]. Importantly, GL-3 resulted accumulated over time in the lysosomes of these cardiomyocytes and typical features of Fabry disease were observed (displacement of cardiac myofibrils to the periphery of the cells, focal areas of myofibrillar lysis, and myofilament degradation with troponin I degradation products). Furthermore, this in vitro model also demonstrated that substrate reduction therapy via inhibition of the enzyme glucosylceramide synthase (GCS) prevented accumulation of GL-3 in hiPSC-CMs.

Since enzyme replacement therapy (ERT) is currently the only efficient therapy in Fabry disease, there is a need to identify pathogenetic biomarkers and therapeutic targets in ERT-treated patients. On this note, Chien and colleagues recently constructed an iPSC-based disease model from patients carrying a *GLA* mutation (IVS4+919 G>A) responsible for Fabry disease [203] and demonstrated for the first time that Interleukin-18 (IL-18), a pro-hypertrophic inflammatory cytokine involved in several cardiac diseases, is involved in the pathogenesis of the disease [204]. Interestingly, these findings suggest that targeting IL-18 might be a potential adjunctive therapy combined with ERT in Fabry patients with the IVS4+919 G>A mutation [204].

#### Diabetes-induced cardiomyopathy

Patients affected by type-2 diabetes mellitus (T2DM) can be more easily affected by coronary artery disease, a condition that can progress to dilated cardiomyopathy and heart failure [205, 206]. Importantly, T2DM alters the cardiomyocyte-metabolic profile [207], which results in the decrease of ATP production followed by reduction of myocardial efficiency and accumulation of toxic lipid metabolites [208]. Furthermore, mitochondrial dysfunction and ROS production activate ROS-sensitive proteases that cleave myofilament proteins [209], whereas proteolytic damage and inadequate protein production cause sarcomere disorganization [66].

In 2014, Drawnel and colleagues investigated diabetes-dependent changes in cardiomyocyte functionality by developing an in vitro DCM model using T2DM-hiPSCs [66]. In such study, the diabetes-induced cardiomyopathy phenotype was recapitulated in hiPSCs-CMs after exposure of the cells to a diabetic environment, consisting on persistent insulin signaling in the absence of glucose, to force the adaptation to fatty acids. Treated cells showed disorganized sarcomeres, altered Ca^2+^ transients, cellular hypertrophy, lipid intracellular accumulation, oxidative stress, and decreased expression of genes controlling protein production. Moreover, treated cardiomyocytes were exposed to a library of 480 compounds to identify small molecules that could prevent the development of the diabetic phenotype. Interestingly, small molecules involved in Ca^2+^ homeostasis and Na^+^ and K^+^ channel blockers, as well as multikinase inhibitors and protein synthesis inhibitors were identified as candidate protective drugs from diabetes-induced cardiomyopathy [66].

### Non-cardiovascular diseases with cardiac traits

#### Duchenne muscular dystrophy

Duchenne muscular dystrophy (DMD) is an X-linked genetic disease caused by frameshift mutations in the *dystrophin* gene, which results in the translation of a truncated and non-functional dystrophin protein [210]. Dystrophin is part of the dystrophin–glycoprotein complex, which connects the actin cytoskeleton to the extracellular matrix, providing cellular stability [211]. In patients affected by DMD, myocytes are particularly sensitive to mechanical stress and rupture, which contributes to muscle degeneration, fibrotic tissue deposition, and premature death. Patients affected by DMD display diastolic dysfunction, arrhythmias, and cardiomyopathy [212].

In 2015, Lin and colleagues generated hiPSC-CMs from healthy individuals and patients affected by DMD. Notably, DMD-hiPSC-CMs recapitulated key features of the disease phenotype (dystrophin deficiency, cytosolic Ca^2+^ overload, mitochondrial damage, and cell apoptosis). Moreover, this study showed that the membrane sealant Poloxamer 188 can suppress the cytosolic Ca^2+^ overload, repress Caspase-3 activation, and decrease cardiomyocyte apoptosis in DMD-hiPSC-CMs [213].

To detect cell structure- and contractile function-properties typical of the DMD disease phenotype, Macadangdang and colleagues cultured healthy and diseased DCM-hiPSC-CMs on a novel engineered platform termed “anisotropically nanofabricated substrata” [212]. This nanopatterned model consisted of 800 nm parallel arrays of grooves and ridges for mimicking the structure of the myocardial extracellular matrix. Although structural differences between healthy and DMD-hiPSC-CMs were masked on the conventional flat substrates, DMD-hiPSC-CMs cultured on the nanotopographic substrate displayed lower structural and functional responses to the underlying nanotopography when compared to healthy cardiomyocytes, probably due to a lower level of actin cytoskeleton turnover, suggesting that DMD-hiPSC-CMs are less adaptable to changes in their extracellular environment [212].

#### Familial transthyretin amyloidosis

Familial transthyretin amyloidosis (ATTR) is a lethal, autosomal-dominant disorder caused by single base-pair mutations in the *TTR* gene encoding for the 55 kDa transport protein transthyretin secreted by the liver [214]. However, the liver is not a clinically relevant site of amyloid deposition in vivo, whilst the brain and the heart are the major organs that are affected, suggesting a need for a multi-lineage model capable of recapitulating the complexity of ATTR disease phenotype in vitro. To model the three major tissues involved in this disease, Leung et al. generated ATTR patient-specific hiPSCs and differentiated them into hepatocytes, neurons, and cardiomyocytes [215]. hiPSC-derived neurons and cardiomyocytes displayed oxidative stress and increased cell death when exposed to TTR produced by patient-matched hiPSC-derived hepatocytes. Moreover, small molecule stabilizers of TTR, such as diflunisal and flufenamic acid, confirmed their efficacy in this model. Collectively, this study recapitulated key aspects of the ATTR disease phenotype in vitro, demonstrating that hiPSCs can also model disorders in which multiple tissues are affected [215].

## Conclusions

hPSC-CMs already have diverse applications, ranging from studying human heart development to cardiac disease modeling and drug testing. They are perceived as having significant value. However, before the technology becomes widely accepted in the cardiovascular disease field as clinically relevant and predictive in human drug testing applications, some crucial hurdles need to be addressed. First, directed differentiation of hPSCs in vitro to specific cardiomyocyte subtypes is still somewhat of a challenge, even though a number of studies have reported specific derivation of atrial-, ventricular-, and pacemaker-like cells. This is due to the limited understanding of later cardiac development in vivo, sometimes continued use of poorly defined (serum-containing) or uncontrolled (such as growth factors not optimally titrated) differentiation culture conditions in vitro. Nevertheless, increased knowledge of heart formation together with deeper understanding of signaling pathways involved in cardiomyocyte development is now leading to the establishment of more defined methods for differentiation that are applicable over multiple hPSC lines and enrich for specific cardiomyocyte subtypes [29, 58, 65, 216–219]. Second, in most standard culture conditions, hPSC-CMs do not display all of the morphological and functional characteristics of adult cardiomyocytes. This needs to be taken into account when studying late-onset cardiovascular diseases but also mechanisms that are based on the highly specialized contraction machinery or gene splicing variants only expressed postnatally. Of note in this context, recent strategies based on biochemical, molecular, or bioengineering approaches [220, 221] have been developed to enhance hPSC-CM maturation. In the biochemical approaches, hormones or adrenergic agonists have been added to improve cardiomyocyte functionality [222]. In the molecular approaches, cardiac ion channels (such as *I*_K1_) and microRNAs have been overexpressed to improve electrophysiology and Ca^2+^ handling [57, 72, 73, 223–225]. In the bioengineering approaches, controlled substrate stiffness, topography, and electrical/mechanical conditioning, as well as integrated systems to deliver nutrients, such as microsystems and bioreactors, all improved sarcomeric organization and contractility [220]. In this regard, additional signatures based on gene expression switches during heart development have been used to track the maturation status of hiPSC-CMs [226]. Among these, inactivation of the fetal *TNNI1* isoform and its replacement by the adult *TNNI3* isoform have proven valuable in quantifying cardiomyocyte maturation in differentiated cultures [46, 226]. Third, the 2D microenvironment in which hPSC-CMs are cultured does not entirely recapitulate the complex dynamics and properties of the human heart [34]. hPSC-CMs can be cultured in 3D either on scaffolds that serve as a platform for cell attachment [227], or in scaffold-free systems in which cells self-organize into structures termed “cardiac microtissues” [228–230]. In this context, several microphysiological systems that use hiPSC-CMs have been developed for drug screening and cardiotoxicity testing [34]. Finally, it is becoming clear that including non-cardiomyocyte cell types to generate multicellular in vitro tissues is essential to advance current disease models, which primarily focus on monotypic cultures of cardiomyocytes, neglecting other cellular components of the myocardium. Endothelial cells, cardiac fibroblasts and smooth muscle cells all provide essential contributions to myocardial structure and function and also play crucial roles in drug-induced cardiovascular toxicity [229, 231]. Providing a system that more closely approximates human heart biology and physiology will allow the generation of more efficient and predictive platforms for modeling complex diseases, for the development of new drug candidates, and also for rescuing (or rehabilitating) molecules that have been withdrawn because of negative outcomes in toxicity assays.

In conclusion, the past few years have witnessed remarkable advances in developmental biology, cell reprogramming, tissue engineering techniques, and in the establishment of innovative molecular assays. Patient-specific hiPSC-CMs and tissue models hold the potential to further advance basic research, on one hand, and personalized and regenerative medicine, on the other hand.

## Abbreviations

AAV: Adeno-associated virus
ALC-1: Atrial myosin essential light chain
ALDH2: Aldehyde dehydrogenase-2
ALPK3: α-kinase-3
AMPK: AMP-activated protein kinase
AP: Action potential
APA: Action potential amplitude
APD: Action potential duration
ARVC: Arrhythmogenic right ventricular cardiomyopathy
ATTR: Familial transthyretin amyloidosis
BrS: Brugada syndrome
BTHS: Barth syndrome
Ca^2+^: Calcium
CAD: Coronary artery disease
CaM: Calmodulin
CaMKII: Ca^2+^/calmodulin-dependent serine–threonine protein kinase II
cAMP: Cyclic adenosine monophosphate
CASQ2: Calsequestrin-2
CDI: Ca^2+^/CaM-dependent inactivation
CFCS: Cardiofaciocutaneous syndrome
CPVT: Catecholaminergic polymorphic ventricular tachycardia
cTnT: Cardiac troponin T
DADs: Delayed after depolarizations
DCM: Dilated cardiomyopathy
DMD: Duchenne muscular dystrophy
EBs: Embryoid bodies
ECC: Excitation–contraction coupling
ECG: Electrocardiogram
EHTs: Engineered heart tissues
ERT: Enzyme replacement therapy
FDA: Food and drug administration
GAA: Acid α-glucosidase
GCS: Glucosylceramide synthase
GL-3: Globotriaosylceramide
HCM: Hypertrophic cardiomyopathy
hECT: Human engineered cardiac tissue
hERG: Ether-a-go-go related gene
hESCs: Human embryonic stem cells
hiPSCs: Human-induced pluripotent stem cells
HLHS: Hypoplastic left heart syndrome
hPSC-CMs: Human pluripotent stem cell-derived cardiomyocytes
IHD: Ischemic heart damage
IL-18: Interleukin-18
iPSCs: Induced pluripotent stem cells
JLNS: Jervell and Lange-Nielsen syndrome
K^+^: Potassium
LAMP2: Lysosomal-associated membrane protein type 2
LQTS: Long-QT syndrome
MCB: Membranous cytoplasmic body
MHC: Myosin heavy chain
MI: Myocardial infarction
MLC: Myosin light chain
Na^+^: Sodium
NCX: Na^+^/Ca^2+^ exchanger
PD: Pompe disease
PGD: Preimplantation genetic diagnosis
PKA: Protein kinase A
PKP2: Plakophilin-2
PPAR-γ: Peroxisome proliferator-activated receptor-γ
RBM20: RNA-binding motif protein 20
rhGAA: Recombinant human GAA
RMP: Resting membrane potential
ROS: Reactive oxygen species
RYR2: Ryanodine receptor-2
SERCA2a: SR Ca^2+^-ATPase
SR: Sarcoplasmic reticulum
TAZ: Tafazzin
TECRL: *Trans*-2,3-enoyl-CoA reductase-like
TS: Timothy syndrome
TTNtvs: TTN-truncating variants
TTX: Tetrodotoxin
T2DM: Type-2 diabetes mellitus
V_max_: Maximum upstroke velocity
